# Tailored Supersaturable Immediate Release Behaviors of Hypotensive Supersaturating Drug-Delivery Systems Combined with Hot-Melt Extrusion Technique and Self-Micellizing Polymer

**DOI:** 10.3390/polym14224800

**Published:** 2022-11-08

**Authors:** Huan Yu, Yinghui Ma, Yanfei Zhang, Huifeng Zhang, Lili Zuo, Chengyi Hao, Weilun Yu, Xiaoying Lin, Yong Zhang, Xianrong Qi, Nianqiu Shi

**Affiliations:** 1 School of Pharmacy, Jilin Medical University, Jilin 132013, China; 2 School of public health, Jilin Medical University, Jilin 132013, China; 3 Department of Biomedical Engineering, Jilin Medical University, Jilin 132013, China; 4 School of Life Science, Jilin University, 2699 Qianjin Street, Changchun 130012, China; 5 Department of Pharmaceutics, School of Pharmaceutical Science, Peking University, Beijing 100191, China; 6 College of Pharmaceutical Sciences, Yanbian University, Yanbian 133002, China

**Keywords:** hypotensive supersaturating drug-delivery systems, amorphization and supersaturation, hot-melt extrusion technique, self-micellizing polymer strategy, tailored supersaturable immediate-release behaviors

## Abstract

The short-term immediate release of supersaturated drug-delivery systems (SDDSs) presents an interesting process that can be tailored to multi-stage release events including initial release after dosing and dissolution, evolved release over longer dissolution periods for biological absorption, and terminal release following the end of immediate release. However, although comprehensive analysis of these critical release behaviors is often ignored yet essential for understanding the supersaturable immediate-release events for supersaturable solid formations when employing new techniques or polymers matched to a particular API. Hot-melt extrusion (HME) has become a popular continuous thermodynamic disordering technique for amorphization. The self-micellizing polymer Soluplus^®^ is reported to be a potential amorphous and amphiphilic graft copolymer frequently used in many nano/micro supersaturable formulations. Our current work aims to develop hypotensive supersaturating solid dispersion systems (faSDDS_HME_) containing the BCS II drug, felodipine, when coordinately employing the HME technique and self-micellizing Soluplus^®^, and to characterize their amorphization as well as immediate release. Other discontinuous techniques were used to prepare control groups (faSDDS_SE_ and faSDDS_QC_). Tailored initial/evolved/terminal three-stage supersaturable immediate-release behaviors were identified and possible mechanisms controlling the release were explored. HME produced the highest initial release in related faSDDS_HME_. During the evolved-release period, highly extended “spring-parachute” process was found in HME-induced amorphization owing to its superior supersaturation duration. Due to the enhanced crystallization inhibition effect, faSDDS_HME_ displayed the strongest terminal release as measured by solubility. For release mechanisms associated with HME, molecular interaction is not the likely dominant mechanism responsible for the improved properties induced by faSDDS_HME_. For release mechanisms involved with the polymer Soluplus^®^ itself, they were found to inhibit drug recrystallization, spontaneously solubilize the drug and lead to improved molecular interactions in all SDDS systems, which were the factors responsible for the improved release. These mechanisms play an important role for the generation of an extended multi-stage immediate release produced via HME or self-micellizing polymer. This study provides a deeper understanding on amorphization and superior multi-stage supersaturable immediate-release behaviors for a particular hypotensive supersaturated delivery system combined with an HME-based continuous manufacturing technique and self-micellizing polymer strategy.

## 1. Introduction

To improve drug solubility, one popular strategy has been to employ supersaturating drug-delivery systems (SDDS) based on the creation of a so-called state of supersaturation [1]. In fact, most active pharmaceutical ingredients (APIs) exhibit poor water solubility and high intestinal permeability, leading to their classification in the Biopharmaceutical Class System (BCS) II drug group. Thus, enhancing solubility to increase BCS II drug bioavailability remains a pivotal challenge facing the pharmaceutical industry [2,3,4]. Toward this goal, the amorphous solid dispersion strategy, as one type of SDDS, is frequently applied to induce the creation of an amorphous drug form existing in a supersaturated state which allows for faster drug dissolution and enhanced bioabsorption [5,6]. Due to the thermodynamic instability of a supersaturated state, which favors crystallization, amorphous polymers were adopted to maintain the supersaturated state and delay/prevent drug crystallization. In supersaturable drug formulations, a thermodynamically unstable and supersaturated solution of a drug is often produced as a higher energy form (referred to as a “spring”). In order to maintain the drug under a state of supersaturation, precipitation or crystallization inhibitors (referred to as the “parachute”) can be added to the system to inhibit or delay crystallization [7,8,9]. In the “spring-parachute” concept, the design focus of SDDS is to enhance the dissolution rate, increase the maximum generated supersaturation, and prolong its duration or maintenance following dissolution by retarding the crystallization of supersaturable drug solutions. Ultimately, it is critically important to identify the “spring-parachute” process associated with a given SDDS in order to understand how best to sustain the supersaturated state.

Short-term immediate release represents the opposite process of long-term controlled release. Differing from long-term controlled release which occurs over a prolonged period (e.g., 1–2 months) [10], the drug is released rapidly and requires a rising drug release at higher concentrations during the limited release period [11]. Supersaturation-induced immediate release is affected by many factors and differs from the common immediate-release process via a unique release mechanism [12]. The height of initial release depends on the supersaturation degree due to amorphization and the formation of a “spring”. Subsequently, the instability of the supersaturable state leads to crystallization and possible decline in the release rate [13,14]. The release evolution is influenced by the interplay and competition between dissolution and crystallization, and causes the “spring-parachute” release kinetics [15]. Following the end of immediate release, the rate of release decreases to the lowest level due to the weakened supersaturation and the tendency of recrystallization [16]. In summary, the entire process regarding the short-term immediate drug release of SDDSs can be tailored to multi-stage release events including initial release after dosing and dissolution, evolved release over a longer dissolution period for biological absorption, and terminal release occurring at the end of immediate release. However, comprehensive analysis of these critical release behaviors is often ignored yet essential for understanding supersaturable immediate-release events for supersaturable solid formations when employing new techniques or polymers as well as the particular BCS II drug.

New strategies and techniques have attracted more attention for the development of SDDS and could significantly impact immediate-release behaviors. The self-micellizing solid dispersion strategy, a promising approach for generating SDDS, was developed in previous studies by some groups based on the use of amphiphilic polymers that can form nano- or micro-scale micelles in aqueous solution through a self-micellizing process [17,18,19,20]. These self-micellizing polymers include Soluplus^®^, poly [MPC-co-BMA], Kolliphor^®^ RH40 and so on. Notably, Soluplus^®^ is reported to be a novel and potential amorphous and amphiphilic polymer composed of polyvinyl caprolactam-polyvinyl acetate-polyethylene glycol graft copolymer. Moreover, Soluplus^®^ has already been shown to have beneficial bifunctional properties as a matrix material and an efficient solubilizer and is viewed as a promising polymer for use in producing a new generation of amorphous self-micellizing systems. More specifically, Soluplus^®^ characteristics (e.g., cloud points) were influenced by dissolution media and its SDDS significantly extended the dissolution/solubility of loaded drugs. In general, many recently reported manufacturing techniques for SDDS generation have been based on strategies employing thermodynamic and kinetic disordering processes [21]. The thermodynamic disordering approach adopts a thermodynamically stable non-crystalline form of a drug as a starting point (e.g., melt and solution) that is subsequently subjected to rapid cooling that induces precipitation of the drug from the solution [22]. By comparison, the kinetic disordering approach is associated with direct solid-state conversion of the crystalline drug into an amorphous state using a mechanical ball-milling or cryo-milling technique [23]. Generally, both disordering strategies have been employed to fabricate SDDSs. However, hot-melt extrusion (HME) has become a highly popular continuous thermodynamic disordering technique globally. HME offers many advantages including its anhydrous participation, solvent-free profile in addition to being a continuous process. Better content uniformity can be obtained by intimate and efficient drug-carrier mixing based on controlling external parameters [24]. Nevertheless, relative to kinetic disordering processes, HME-based continuous thermodynamic disordering manufacturing technology holds promise as an extremely suitable strategy for the industrial-scale generation of SDDSs [25].

In modern society, cardiovascular disease is a widespread and significant contributor to morbidity and mortality especially in the middle-aged and elderly populations. It is well known that cardiovascular disease commonly accompanies hypertension that is related to a greater incidence of heart failure and death [26]. Therefore, the prevention and treatment of hypertension are vital for the control of cardiovascular disease, which in turn leads to reduced healthcare system costs and improved quality of life. Felodipine (FEL), a dihydropyridine calcium channel blocker, is a therapeutic agent used for the treatment of hypertension. As a Biopharmaceutical Class System (BCS) II drug (low solubility and high permeability), FEL is a highly lipophilic entity (oil/water partition coefficient log *P* value of 4.46 [27], where log *P* represents lipophilicity) and is practically insoluble in aqueous gastrointestinal fluids. Such poor aqueous solubility weakens oral absorption of FEL, resulting in low bioavailability and high variability in systemic exposure after oral administration. Ultimately, the enhanced hypotensive efficacy of FEL in vivo will require improvements in its solubility and dissolution properties and extension of immediate release behaviors.

**Figure 1:**
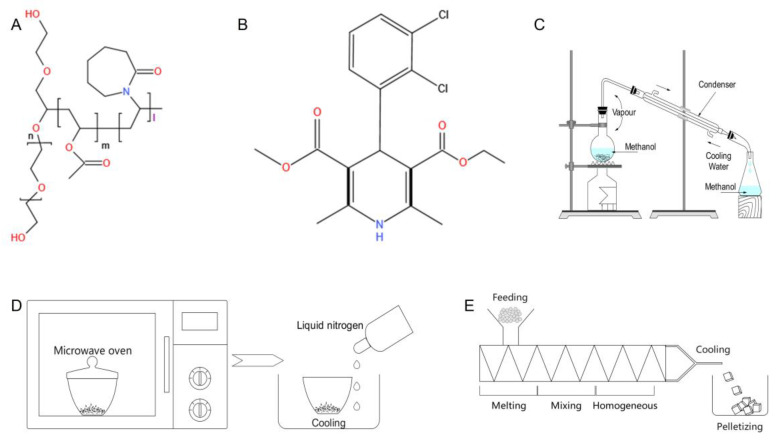
(**A**,**B**) Chemical structure of self-micellizing polymer Soluplus^®^ (**A**) and hypotensive BCS II drug FEL (**B**). (**C**–**E**) Fabrication techniques including discontinuous thermodynamic disordering approaches (solvent evaporation (**C**) and microwave irradiation-quench cooling (**D**)) and continuous thermodynamic disordering approach ((**E**), hot-melt extrusion).

To our knowledge, the tailored immediate-release behaviors of felodipine-based hypotensive supersaturating drug-delivery systems employing a continuous thermodynamic disordering manufacturing technique and self-micellizing strategy remain unclear and warrant further exploration. Here, we employed hot-melt extrusion (HME) as a continuous thermodynamic disordering approach, and two controlled methods including solvent evaporation and microwave-quench cooling as discontinuous processes. These methodologies were chosen to fabricate amorphous supersaturating drug-delivery systems containing the self-micellizing polymer Soluplus^®^ for the supersaturating immediate-release of felodipine, a water-insoluble BCS II antihypertensive drug (Figure 1A–E). Felodipine-based amorphous supersaturating drug-delivery systems were developed using HME (faSDDS_HME_) and other control techniques (faSDDS_SE_ and faSDDS_QC_). Their amorphization characterization and solid-state properties were evaluated via scanning electron microscopy, powder X-ray diffraction, and differential scanning calorimetry. To identify the tailored immediate-release behaviors, first, dissolution profiles were measured in various media environments to clarify the initial release behaviors systematically. Second, for monitoring the evolved release mechanism, “spring-parachute” processes for each faSDDs system were investigated via semi-continuous determinations followed by quantification of results in order to elucidate molecular mechanisms responsible for the observed supersaturation states, since little is known about the “spring-parachute” processes occurring during the evolution of supersaturation in a liquid environment. Third, terminal release was reflected by the measurement of solubility in different media environments at the end of 24 h or 48 h testing periods. In addition to identify these comprehensive release behaviors, other experiments were conducted to understand the properties of faSDDSs and superior immediate-release characteristics offered by the HME technique. Stability of the amorphous state in a solid environment during the “storage” stage was assessed after being kept in storage for 1, 3 and 4 months. Moreover, underlying mechanisms involved in the formation of faSDDSs systems themselves or superior faSDDS_HME_ were explored by investigating molecular interactions, crystallization inhibition effects and solubilization effects via Fourier transform infrared spectroscopy (FT-IR), simulated crystallization assay and phase solubility measurements, respectively. Generally, the results of this work facilitate the development and industrialized application of felodipine-based hypotensive amorphous supersaturating drug-delivery systems combined with extrusion technique and self-micellizing strategy from the perspective of tailored supersaturable immediate-release events.

## 2. Materials and Methods

### 2.1. Materials

Felodipine (purity > 99%, Mw 384.25) was obtained from Kangbaotai Fine Chemicals Co., Ltd. (Wuhan, China). BASF (Ludwigshafen, Germany) generously sponsored the polyvinyl caprolactam-polyvinyl acetate-polyethylene glycol graft copolymer (Soluplus^®^, Mw 90,000–140,000). Methanol, hydrochloric acid, potassium dihydrogen phosphate, and sodium hydroxide (analytical grade) were purchased from Tianjin Damao Chemical Reagent Factory. Figure 1 shows the chemical structures of the drug and polymer.

### 2.2. Development of Various faSDDS Systems Based on Hot-Melt Extrusion Technique and Other Discontinuous Approaches

#### 2.2.1. Fabrication of faSDDS_HME_ Based on Hot-Melt Extrusion Technique

To formulate faSDDS_HME_ using the HME method, briefly, we used a hot-melt extruder (Pharma 11; Thermo Fisher Co., Shanghai, China) and mixed FEL with Soluplus^®^ in *w*/*w* ratio of 1:4 (drug: carrier) in the feeding apparatus. Afterward, the obtained mixture was extruded by using the co-rotating twin-screw of the extruder under a screw rate of 70 rpm and extrusion temperature of 125 °C to produce faSDDS_HME_ exudates that were then cooled at ambient temperature. Next, we milled the produced extrudates using a laboratory mixer. The extrudates were then passed through an 80-mesh sieve, and subsequently stored in an air-tight container under ambient conditions before experimentation.

#### 2.2.2. Fabrication of faSDDS_SE_ Based on Solvent Evaporation Technique

Soluplus^®^ carrier was added and mixed with FEL with a ratio of 1:4 (drug: carrier = *w*/*w*). Afterward, the mixture was dissolved in methanol, and the produced solvent was evaporated at 40 °C. Next, the generated samples were placed in a vacuum dryer for 24 h to completely evaporate the solvent. The dried samples were passed through the 80-mesh sieve, and then stored in a desiccator before experimentation.

#### 2.2.3. Fabrication of faSDDS_QC_ Based on Microwave Irradiation-Quench Cooling Technique

A microwave-activated solid dispersion comprising a 1:4 *w*/*w* ratio of FEL: Soluplus^®^ was obtained via microwave irradiation and designated faSDDS_QC_. Next, a certain amount of the physical mixture containing the polymer and drug was removed, transferred to a glass beaker and then placed under microwave irradiation for 2 min at the selected power level of 700 W in a P70F23P-G5(SO) microwave oven (Galz, Guangzhou, China) to produce the homogeneous fused liquid. Thereafter, liquid nitrogen was introduced to quickly cool the fused liquid at −196 °C to produce a crude preparation at faSDDS level. After that, the produced crude faSDDS_QC_ preparation was then ground in a glass mortar, and passed through an 80-mesh sieve to finally obtain particles of uniform size. Before experimentation, each faSDDS_QC_ powder was stored in a desiccator.

#### 2.2.4. Preparation of Physical Mixtures and Pure Amorphous FEL

In order to produce the physical mixture (PM) of FEL and polymer (FEL_PM_), we vigorously mixed FEL with Soluplus^®^ at a *w*/*w* ratio of 1:4 (drug: carrier) for 10 min in a plastic bag. To generate pure amorphous FEL (FEL_PA_), FEL was placed in preheated porcelain crucibles, and then heated over a dimethicone bath to both promote fusion (170 °C) and keep the FEL in a homogenous liquid state for 5 min. After that, we quench-cooled the fused liquid by rapid cooling using liquid nitrogen at −196 °C, followed by immediate storage in a desiccator to prevent moisture absorption at room temperature. Next, we pulverized the dried powders using a mortar and pestle, and then filtered the powders through an 80-mesh sieve to produce pure amorphous FEL powder. The pure powder was stored in a desiccator before the subsequent experiments.

### 2.3. Solid-State Characterization

#### 2.3.1. Scanning Electron Microscopy (SEM) Experimentation

Surface morphologies of various faSDDS systems and control groups were determined using SEM (JSM-6490LV, JEOL, Tokyo, Japan). Each sample was prepared by affixing the dried powder to a glass stub sample holder using double-sided adhesive tape. Next, samples were coated with gold to make them electrically conductive. Images were captured using an accelerated voltage of 20.0 kV and then were analyzed to detect changes in the polymorphic state.

#### 2.3.2. Powder X-ray Diffraction (PXRD)

The PXRD pattern for various faSDDS systems and controlled samples was obtained within the 2θ range of 5°~45° at a scanning speed of 10°/min and a step size of 0.04° using an X-ray power diffractometer with a Cu-Kα radiation (λ = 1.541 Å) source (Rigaku, Tokyo, Japan). A tube voltage of 40 kV and amperage of 40 mA were used.

### 2.4. Initial Release in Various Media Environments Measured by Dissolution

ZRS-8G dissolution apparatus (Tianda, Tianjin, China) was used to assess the dissolved satiation of FEL in each sample. After accurately weighting 30 mg of the FEL in faSDDS system, we placed the powders in vessels containing 900 mL dissolution medium (PBS pH 4.5, 6.8, 7.5 or 0.1 M HCl). During the experimental process, we controlled the temperature at 37 ± 0.5 °C and set the rotating speed of the paddle at 100 rpm. An aliquot of 5 mL was removed from each solution at different time points of 5, 10, 15, 30, 60, 90 and 120 min, and then each sample was added with the equal volume of fresh medium. The 0.45 μm Millipore filters was used to filter sample solutions. UV–visible spectroscopy was used to assess FEL content at 360 nm. Samples were prepared in triplicate during experiments.

### 2.5. Evolved Release during Longer Dissolution Period Monitored by “Spring-Parachute” Process

#### 2.5.1. Semi-Continuous Determinations

“Spring-parachute” profiles were obtained via long-term (36 h) collection of dissolution data from samples and controls. PBS (900 mL, pH 6.8) was chosen to dissolve various faSDDS systems and controlled samples containing equivalent FEL amounts (30 mg FEL), and the mixture was then placed in the dissolution apparatus. After that, an aliquot was removed from each solution every 0.5 h (n = 1) over a period of 36 h.

#### 2.5.2. Process Quantification

The samples obtained in Section 2.4 were filtered using 0.45-μm Millipore filters and then the FEL content in each filtrate was determined using UV–visible spectroscopy at 360 nm. Next, the “spring and parachute” curves were assessed using the collected data. Corresponding parameters were calculated or simulated in order to quantify the “spring-parachute” process.

### 2.6. Terminal Release in Various Media Environments Measured by Solubility

Equilibrium solubilities were measured in 0.1 mol/L HCl and PBS solutions of various pH levels (pH 4.5, 6.8, 7.5). Each glass vial was added with an excess amount of sample containing 50 mL of the abovementioned solutions. Next, the vials were sealed and later placed in an oscillator at 37 °C for 24 h and 48 h. Afterward, the 0.45-μm Millipore filters were used to filter the samples. A UV–VIS spectrometer (UV-1800, Shimadzu, Japan) was used to analyze the filtered samples at λ_max_ 360 nm. Calibration curve was linear in the concentration range of 0.80 μg/mL-50 μg/mL with R^2^ > 0.9995 and equation of y = 0.0188x + 0.0003. Samples were prepared and repeated in triplicate during the experimental procedure.

### 2.7. Stability of faSDDS and Possible Mechanisms for Supersaturable Immediate Release

#### 2.7.1. Stability of the Amorphous State

Various faSDDS systems and controlled samples were stored in sealed glass bottles, and kept under dry conditions at a temperature of 25 °C and monitored for crystallization via PXRD at 1, 3 and 4 months of storage. If the diffractogram displayed any peaks, we speculated the occurrence of crystallization events.

#### 2.7.2. Fourier Transform Infrared Spectroscopy (FT-IR)

In order to explore the possible interactions between drug and polymer in faSDDS systems, we mixed the sample (2–3 mg) with dry potassium bromide, and then compressed the mixed powders into a pellet using a powder-compressing instrument. FT-IR experiments were carried out using an Agilent Cary 660 FTIR Spectrometer (Nicolet IS5, Thermo Scientific, Waltham, MA, USA) to evaluate the spectrum of each sample. The spectrum for each sample was collected in the 400–4000 cm^−1^ range under a resolution of 1 cm^−1^.

#### 2.7.3. Simulated Crystallization Assay

Simulated crystallization assay was performed to evaluate the crystallization inhibition ability of polymer at various concentrations to maintain FEL supersaturation. Briefly, 900 mL of PBS solution (pH 6.8) containing different concentrations of Soluplus^®^ (0, 0.15, 0.2, 0.25 and 0.3 mg/mL) in a paddle apparatus. The obtained dissolved media were stirred under 100 rpm at 37 ± 0.2 °C. FEL–methanol solution (300 µL, with concentration of 166.6 mg/mL) was added into the dissolution media. Afterward, we removed 5 mL samples at different time points of 15, 30, 60, 90, 120, 150, 180, 210 and 240 min and filtered the collected samples using a 0.45-mm filter. As described in Section 2.4, the FEL concentrations were determined by UV–VIS method (Section 2.4).

#### 2.7.4. Phase Solubility

As previously described [28], phase solubility for aqueous solutions containing increasing Soluplus^®^ concentrations ranging from 0 to 0.9 mg/mL in PBS (pH 6.8) that were added to an excess amount of FEL were measured. Each sample collection was repeated in triplicate. After being sealed, the flasks were shaken for 24 h in constant temperature water bath equipment at 37 °C. Afterward, each sample was filtered by passing through a 0.45-mm filter. The obtained filtered sample was thoroughly diluted. As described in Section 2.4, the concentration of FEL in each sample was determined by UV–VIS at 360 nm.

### 2.8. Data Analysis

Data were marked as the mean ± standard deviation (SD). Statistical analysis of data among different groups was carried out using the unpaired Student’s *t*-test. Each statistical experiment was tested in triplicate (*n* = 3). Data with *p* < 0.01 and *p* < 0.05 levels were chosen as statistically significant.

## 3. Results and Discussion

### 3.1. Fabrication and Characterization of Various faSDDS Systems

**Figure 2:**
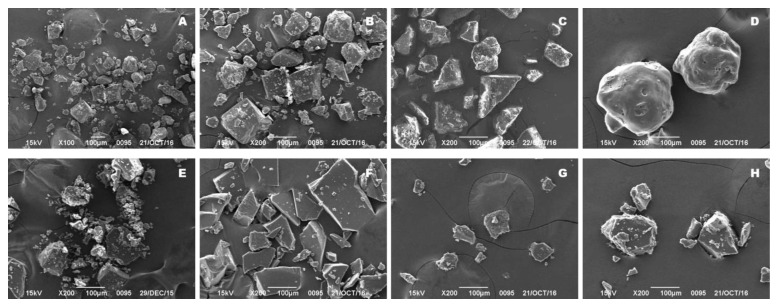
SEM micrographs showing crystalline FEL ((**A**), 100×), crystalline FEL ((**B**), 200×), FEL_PM_ ((**C**), 200×), Soluplus^®^ ((**D**), 200×), FEL_PA_ ((**E**), 200×), faSDDS_SE_ ((**F**), 200×), faSDDS_QC_ ((**G**), 200×), and faSDDS_HME_ ((**H**), 200×).

In the SEM photomicrographs, crystalline FEL appeared as square-shaped crystalline structures (Figure 2A,B), while SEM observations of the physical mixture containing FEL and self-micellizing polymer (Figure 2C) revealed that FEL was present in crystalline form. Meanwhile, the polymer Soluplus^®^ itself presented spheroidal shapes (Figure 2D). There was significant change in the micromorphology of particles in pure amorphous FEL, but a minimal change in faSDDS groups (Figure 2E–H). These observations revealed that the FEL in the faSDDS systems or amorphization alone might exist in an amorphous form, which may be responsible for the change in dissolution rate. However, the use of other techniques is still required for amorphous state verification.

**Figure 3:**
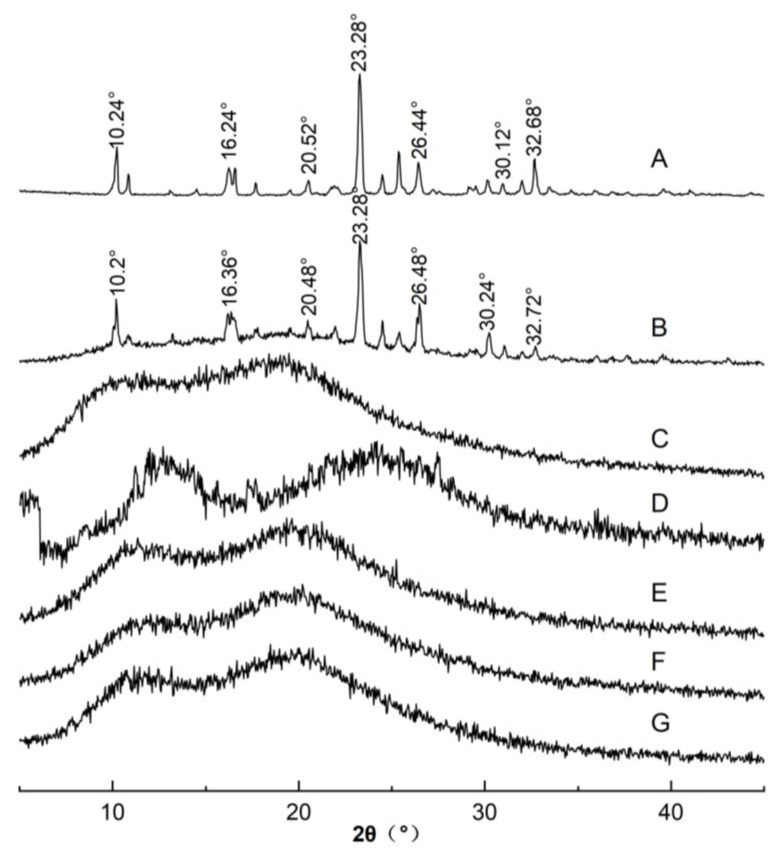
PXRD spectra of FEL (A), FEL_PM_ (B), Soluplus^®^ (C), FEL_PA_(D), faSDDS_SE_ (E), faSDDS_QC_ (F) and faSDDS_HME_ (G).

Hitzer et al. reported that PXRD as a very convenient method in measuring or determining whether materials are in an amorphous state or not [29]. In Figure 3, a series of major diffraction peaks were observed for FEL at 10.24°, 16.24°, 20.52°, 23.28°, 26.44°, 30.12° and 32.68° (Figure 3A), whereas no obvious diffraction peaks were observed for Soluplus^®^ (Figure 3C), probably due to its amorphous nature. After mixing FEL with Soluplus^®^, the PM diffractogram showed clearly all major characteristic crystal peaks of FEL, although the drug peaks were of decreased intensity, presumably due to effects of the interaction between FEL and the excipient (Figure 3B). Meanwhile, the disappearance of the sharp peaks indicated apparent differences between the PXRD profile of FEL_PA_ and the profiles of pure crystalline drugs and physical mixtures.

In contrast, when FEL was amorphized into the Soluplus^®^ polymer carrier using the discontinuous and continuous thermodynamic disordering strategies, the characteristic crystalline FEL peaks were absent in faSDDS_SE_, faSDDS_QC_ and faSDDS_HME_, implying that the transformation from the crystal state to an amorphous state of the drug could be effectively completed by the tested interaction between FEL and excipient. Amorphous FEL, due to its relatively higher energy state, dissolves and releases from solid dispersion excipients more readily than crystalline FEL. In addition, drug–polymer molecule interactions were extended after drug-carrier treatment by an amorphous process compared to PM of FEL_PM_ and Soluplus^®^, as previously reported [30]. Thus, SEM, and PXRD results collectively indicated that a completely amorphous state was achieved when employing the self-micellizing polymer Soluplus^®^ to form amorphous faSDDS delivery systems. Nevertheless, it was unclear whether the amorphous state change of FEL in faSDDS systems could lead to the improvement of immediate release warranting additional studies.

### 3.2. Tailored Supersaturable Immediate-Release Behaviors

#### 3.2.1. Initial Release Behavior via Dissolution Measurement

**Figure 4:**
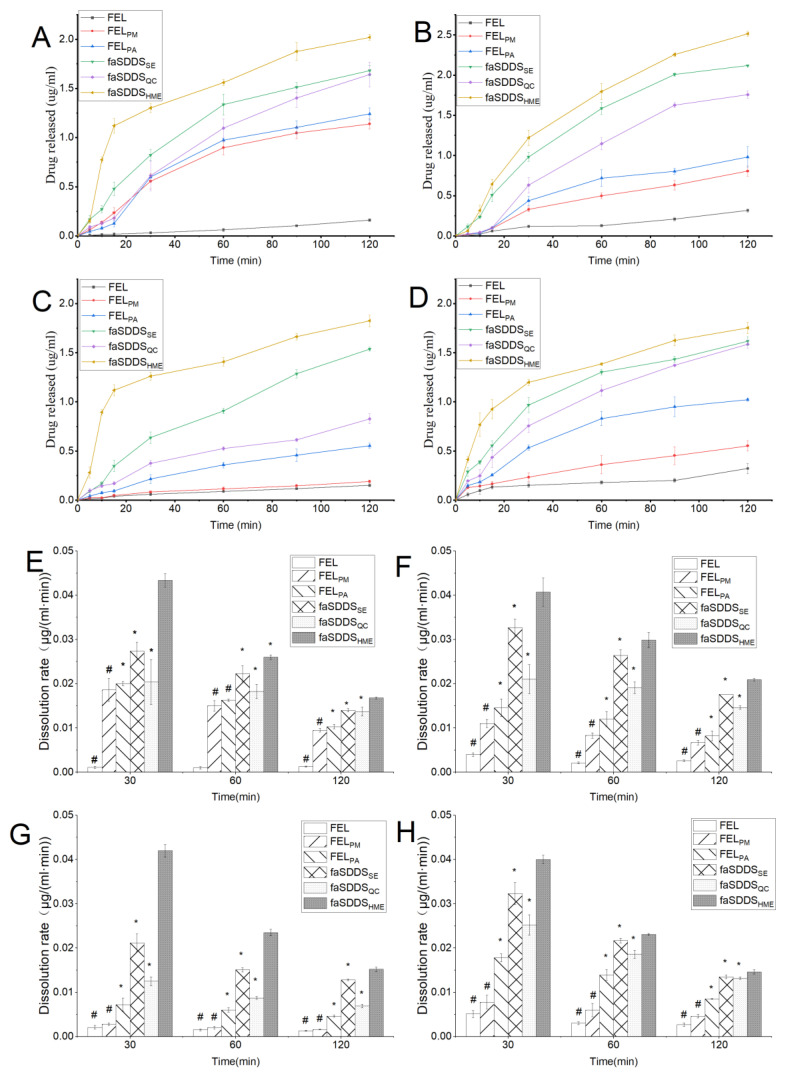
Dissolution profiles of FEL, FEL_PM_, FEL_PA_, faSDDS_SE_, faSDDS_QC_, and faSDDS_HME_ in 0.1 mol/L HCl (**A**), PBS (pH 4.5, (**B**)), PBS (pH 6.8, (**C**)), and PBS (pH 7.5, (**D**)). Dissolution rates were calculated at 30, 60 and 120 min for FEL-related samples in 0.1 mol/L HCl (**E**), PBS (pH 4.5, (**F**)), PBS (pH 6.8, (**G**)), and PBS (pH 7.5, (**H**)). # *p* < 0.05, FEL and FEL_PM_ vs. faSDDS_SE_, faSDDS_QC_, and faSDDS_HME_, * *p* < 0.05, faSDDS_SE_ and faSDDS_QC_ vs. faSDDS_HME_. Standard deviation expressed by error bars (*n* = 3).

Figure 4 shows dissolution profiles over 120 min for crystalline FEL, FEL_PM_, FEL_PA_, faSDDS_SE_, faSDDS_QC_, and faSDDS_HME_. Crystalline FEL exhibited only slightly lower dissolution in 0.1 M HCl, PBS solutions (pH 4.5, pH 6.8, and pH 7.4), while the dissolution of FEL_PM_ increased slightly but not significantly. Notably, pure amorphous drug FEL_PA_ significantly improved the solubility of the crystalline drug, and greater FEL dissolution was obtained using faSDDS systems (faSDDS _SE_, faSDDS _QC_ and faSDDS _HME_) relative to crystalline FEL and FEL_PM_. In fact, faSDDS systems supported more extended dissolution and brought about an obvious dissolution enhancement in the media environments tested here. For example, Figure 4C shows that in PBS (pH 6.8) at 120 min, dissolution amounts of FEL in faSDDS_SE_, faSDDS_QC_ and faSDDS_HME_ were 10.08-, 5.43- and 11.96-times higher than that of its crystal form, respectively. Overall, the analysis results of the dissolution process revealed that the dissolution levels for the three faSDDS systems ranged in order from maximum to minimum: faSDDS_HME_ > faSDDS_SE_ > faSDDS_QC_.

Interestingly, during the 120-min dissolution period, a “spring” phenomenon was observed for the faSDDS such that drug concentrations continued rising during the initial dissolution process. However, no “parachute” effect was observed despite using a prolonged testing time to capture a possible “spring-parachute” profile. In addition, all faSDDS dissolved slowly as a consequence of the high viscosity of Soluplus^®^ in the solution at 37 °C. Previous studies demonstrated that Soluplus^®^ in supersaturated solution promoted drug dissolution and thereby suppressed drug crystallization from the dissolved state through self-micellization. Moreover, the special grafted structure of Soluplus^®^ provided amphiphilicity, which facilitated micelle formation at a critical micelle concentration (CMC) of only 7.6 μg/mL [31]. The results of this research showed that Soluplus^®^ has a higher concentration (about 130 μg/mL) in faSDDS than that of Soluplus^®^ in the CMC, indicating that the solubility of FEL may be steadily improved owing to micelle formation.

The dissolution rates of the faSDDS systems were assessed according to the data collected at the different time points of 30, 60, and 120 min during the experimental process (Figure 4E–H). The calculation of the dissolution rate was based on the method previously reported by Almotairy et al. [32] and Fael et al. [33]. From the results shown in Figure 4E–H, we observed that the dissolution rates for the faSDDS systems were relatively higher at the early stage, but gradually decreased thereafter. The dissolution rate calculated from the data at different time points were ranked as follows: faSDDS_HME_ > faSDDS_SE_ > faSDDS_QC_ > FEL_PA_ > FEL_PM_ > FEL. In fact, dissolution rates for FEL and FEL_PM_ were both dramatically lower than those of the three faSDDS, with faSDDS_HME_ exhibiting a significantly higher dissolution rate than corresponding rates of faSDDS systems prepared via the other two methods. Therefore, the HME-triggered process achieved a significant initial release by the determined greater enhanced dissolution as compared to QC or SE-based processes, deduced from the dissolution curves and dissolution rate assays of FEL-related samples.

Next, the Noyes–Whitney equation (Equation (1)) [34] was introduced to further analyze the above observed significantly enhanced initial-release behavior of the faSDDS system prepared by the HME method during the initial phase of dissolution:

(1) $$\frac{dC}{dt}=kS\left(C_{S}-C\right)=\left(\frac{Ds}{vh}\right)\left(C_{S}-C\right)$$

where the dissolution rate is expressed by *dC*/*dT*, μg/(mL·min), the diffusion coefficient is denoted by *D*, the dissolution rate constant is marked by *k*, the available surface area is represented by *S*, the saturated solubility of drug in the medium is represented by *C_S_*, the selected volume of medium used for dissolution is marked by *v*, the thickness of the diffused layer is represented by *h*, and *C* represents the drug concentration in the bulk fluid at time point *T*. Based on this equation, the dissolution character of the insoluble drug FEL can be expressed to show that the rise in saturation solubility (*C_S_*) of FEL in faSDDS_HME_ samples induced a significant increase in dissolution rate (*dC*/*dT*). In combination with the results of solubility determinations in Section 3.2.3, it can be found that the saturation solubility of faSDDS_HME_ was significantly higher than that of other faSDDS systems. The higher saturation solubility of faSDDS_HME_ is likely responsible for the superior initial drug release performance.

#### 3.2.2. Evolved Release Behavior via Monitoring of the “Spring-Parachute” Process

The supersaturation ratio *S* (Equation (2)) [35] and the relative supersaturation index *σ* (Equation (3)) [35] together decides the state of supersaturation:

(2) S=CCequ

(3) σ=S−1=(C−Cequ)Cequ

where the concentration at time point *T* and the equilibrium solubility are represented by *C* and *C_equ_*, respectively. As described previously, the initial dissolution tendency was enhanced akin to a “spring” during the initial release period [7], while thermodynamic instability of the amorphous form favoring slow drug crystallization functioned akin to a “parachute” [36]. Both processes reflect the concept of “spring-parachute” suggested in previous studies [7,36,37]. For supersaturating amorphous systems, it is important to analyze “spring-parachute” processes to dynamically monitor dissolution and compare release effects of the various thermodynamic disordering approaches. However, with regard to particular hypotensive supersaturating faSDDS systems based on HME technique and self-micellizing polymer Soluplus^®^, current understanding of their evolved release and associated “spring-parachute” process is limited, thus prompting the investigation of these processes in our work.

**Figure 5:**
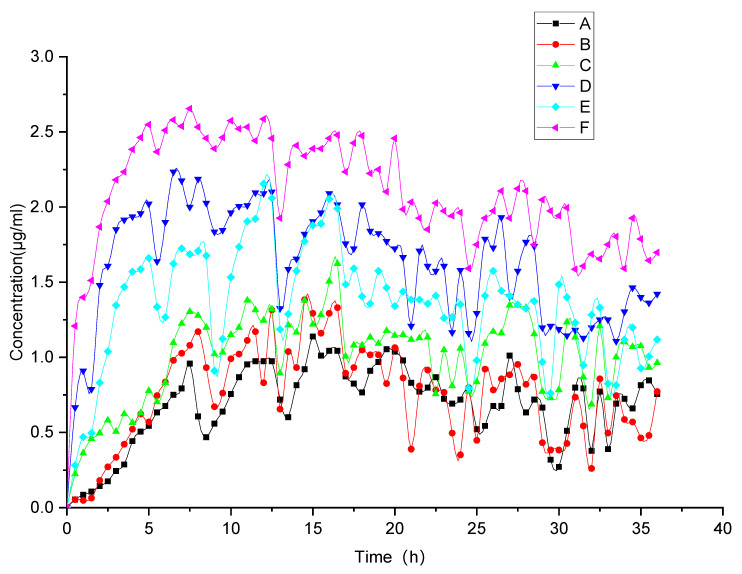
“Spring-parachute” profile of FEL-based samples including FEL (A), FEL_PM_ (B), FEL_PA_ (C), faSDDS_SE_ (D), faSDDS_MC_ (E), faSDDS_HME_ (F) over a 36 h period at the equivalence of 30 mg FEL in PBS (pH 6.8) using dissolution apparatus.

We conducted the evolved release analysis by determining “spring-parachute” process using a semi-continuous monitoring pattern. Figure 5 shows “spring-parachute” curves for all groups with PBS (pH 6.8) as the medium. The curve for FEL continued to rise to a maximum approaching 1.13 μg/mL during the initial 0–6 h, and then flattened somewhat before undergoing a fluctuation after 6 h. The concentration curve for the physical mixture was similar to that obtained for crystalline FEL, but with slightly higher concentration values. For pure amorphous FEL, higher concentration values were obtained relative to values for the crystalline FEL form and the physical mixture due to advantages associated with the amorphization process. Nonetheless, enhancement of the “spring-parachute” effect for amorphous FEL was not dramatic, since amorphization did not always result in significant solubility enhancement, as reported previously [38]. The key parameters and profiles of the “spring-parachute” for comparing faSDDS systems are displayed in Figure 5 and Table 1. During the evolved release period, the total faSDDS systems performed the corresponding higher “spring-parachute” curves relative to those obtained for crystalline and amorphous FEL, with the highest AUC and C_max_ values obtained measuring 70.52 μg·h/mL and 2.65 μg/mL for the faSDDS_HME_ system. Ultimately, AUC and C_max_ values for all faSDDS systems are ranked: faSDDS_HME_ > faSDDS_SE_ > faSDDS_MC_.

#### 3.2.3. Terminal Release Behavior via Solubility Measurement

*SDDS* can store potential energy and form systems similar to “compression spring” systems according to “spring-parachute” theory. When such a system encounters the medium, potential energy is released accompanied by dispersion that enables the molecules to assume a state of supersaturation. Due to the thermodynamically unstable character of supersaturation, such formulations must provide a “parachute” to maintain high concentrations of the soluble drug and to ensure good absorption for biologically relevant timeframes. For creation of a suitable supersaturated system, the ideal carrier should rapidly dissolve and reach an adequately high concentration to effectively maintain the drug in a supersaturated state [39]. Therefore, it is important to evaluate terminal release behavior by measuring solubility to compare the potential advantages of HME process tuning compared to other methods.

**Figure 6:**
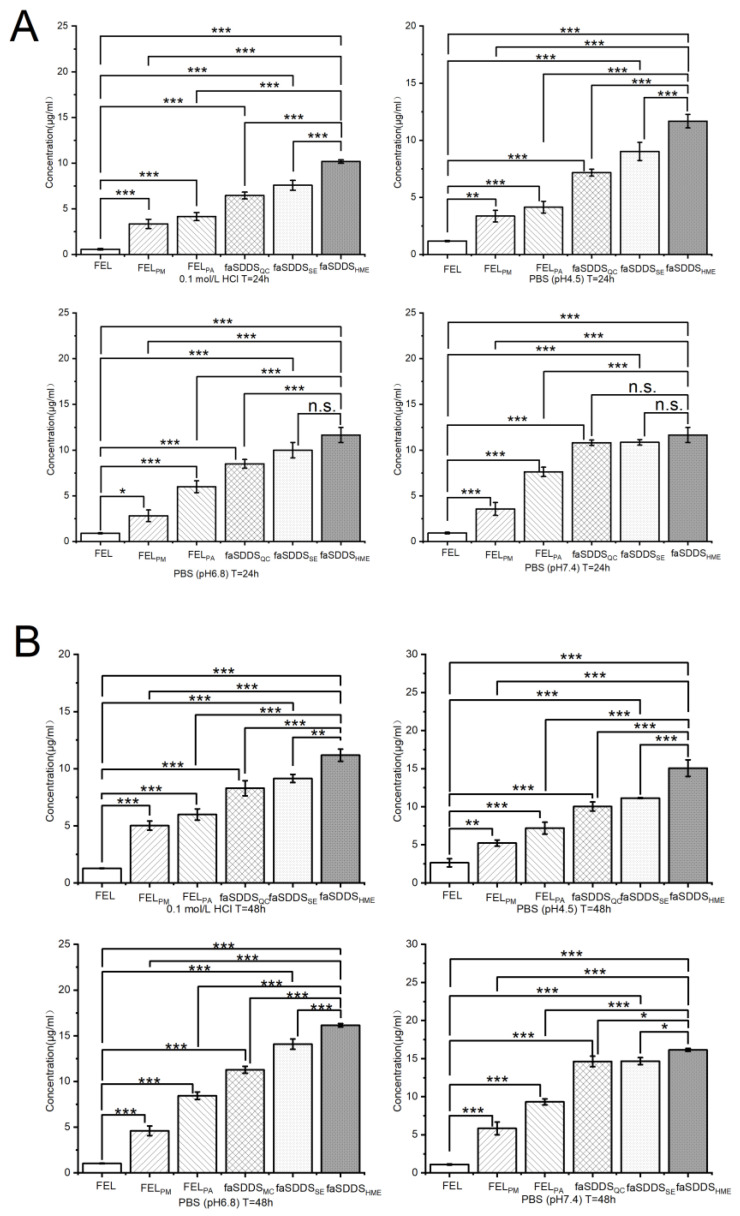
Solubility of samples in different solutions (0.1 mol/L HCl and PBS (pH 4.5, 6.8, 7.5)) at 24 h (**A**) and 48 h (**B**); * *p* < 0.05, ** *p* < 0.01, *** *p* < 0.001, and NS, no significant difference. Standard deviation expressed by error bars (*n* = 3).

As shown in Figure 6, the solubility assay in Figure 6A,B depicted solubility results for FEL-related samples at two time points of 24 h and 48 h, respectively. When FEL was physically mixed with Soluplus^®^, solubility increased, but this increase was weaker than that of faSDDS prepared using Soluplus^®^ as a carrier. Pure amorphous drug FEL_PA_ was effective to improve solubility of crystalline FEL. In the faSDDS generated using different methods, FEL solubility significantly improved in descending order as follows: faSDDS_HME_ > faSDDS_QC_ > faSDDS_SE_. Since FEL is a non-ionic compound, the effects of dissolution media on solubility of FEL-related samples were weak. At 24 h, faSDDS_HME_ exhibited the greatest solubility of around 10 μg/mL, especially in pH 1.0 and pH 4.5 media, with superior solubility improvement observed relative to that provided by faSDDS_QC_ or faSDDS_SE_. Notably, no significant differences in solubility improvement were found for three faSDDS groups in pH 7.4 media after 24 h. However, after 48 h, differences were obvious and faSDDS_HME_ exhibited maximum solubility improvement (about 10–16 μg/mL) in all media regardless of pH, thus indicating that faSDDS_HME_ attained highest solubility at 24 h or 48 h relative to the other faSDDS systems. All of these findings revealed that *SDDS* systems generated via the HME had the unique advantage of improved solubility, resulting in the elevated terminal release.

In summary, the SDDS strategy can significantly enhance the maintenance of the “spring-parachute” process. In this work, the HME process outperformed QC and SE processes by enhancing the FEL initial release (dissolution) and moving up the evolved release (“spring-parachute” process). Moreover, amorphous SDDS generated via the HME demonstrated a stronger ability to delay the “parachute” process, possibly due to its ability to inhibit FEL recrystallization by delaying crystallization onset and subsequent crystal growth. Highly elevated terminal release was found in HME-based faSDDS system via the solubility analysis. HME enhanced the initial release and evolved release of the drug from faSDDS system, which is favorable to elevate terminal release owing to the optimal maintenance of supersaturation. Overall, improved multi-stage release behaviors were found in HME-induced faSDDS. The “spring-parachute” process can be used to monitor the stability of an amorphous drug over a longer dissolution period in a liquid environment. However, for an amorphous system, it is also important to explore drug stability in a solid environment during storage. Therefore, the stability of the amorphous state was assessed via the determination of PXRD profiles. Moreover, relevant mechanisms were also explored to understand the advantage of supersaturable immediate release resulting from faSDDS or faSDDS_HME._

### 3.3. Stability of faSDDSs and Possible Mechanisms Controlling Supersaturable Immediate Release

#### 3.3.1. Stability of Various faSDDS Systems

**Figure 7:**
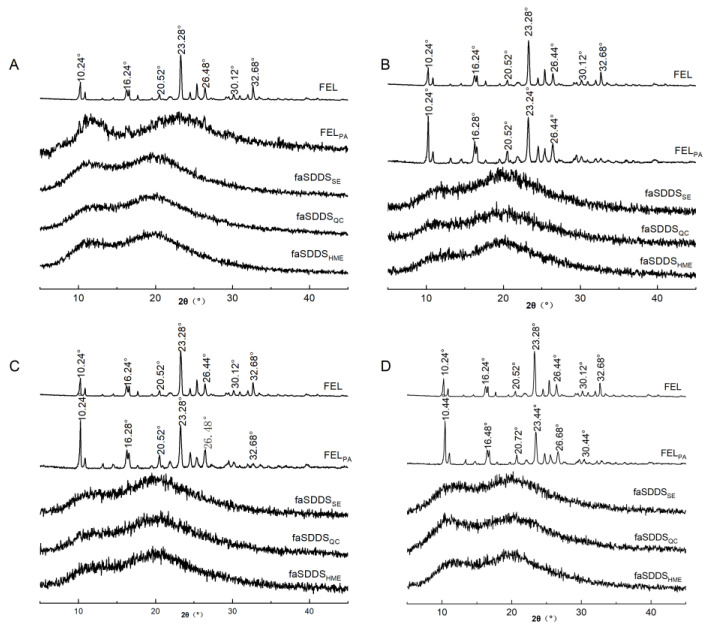
Stability of crystalline FEL, FEL_PA_, faSDDS_SE_, faSDDS_QC_ and faSDDS_HME_ by powder X-ray diffractogram after 0 month (**A**), 1 month (**B**), 3 months (**C**), and 4 months (**D**).

To explore the solid-state stability of the amorphous state in faSDDS systems and compare it with that of a pure amorphous drug, materials were stored at 25 °C under dry conditions and then the physical stabilities of faSDDS and amorphous FEL systems were analyzed using PXRD. The final obtained PXRD results of amorphous faSDDS and amorphous FEL before and after storage are shown in Figure 7. It can be seen from Figure 7A that for each sample no obvious characteristic crystal diffraction peak was observed, indicating that all samples existed in an amorphous state at the beginning of storage (month 0). By contrast, it can be seen from Figure 7B that after 1 month of storage, crystal diffraction peaks appeared that corresponded to amorphous FEL positions of 10.24°, 16.28°, 20.52°, 23.24° and 26.44° as characteristic FEL peaks. This result indicated that pure amorphous FEL in the absence of any *SDDS* process was not stable enough to avoid crystallization after 1 month of storage. More specifically, low T_g_ was proved to be correlated to the instability of amorphous FEL, which was characterized by the positive correlation between rapid molecular movement and immediate FEL crystallization under ambient conditions [40]. Meanwhile, PXRD diffraction peak profiles of faSDDS samples stored for additional time periods of 3 and 4 months were similar to corresponding profiles obtained at the beginning of storage (Figure 7C,D). Thus, these patterns contained no FEL diffraction peaks and thereby proved that the amorphous drug possessed relatively good physical stability because it did not crystallize in the SDDSs. Laitinen and colleagues proved that the stability of the amorphous drugs could be maintained by excipients through increasing the glass transition temperature (T_g_) of the systems, followed by decreasing their molecular mobility in solid dispersion systems [40]. In addition, another important stabilization mechanism underlying inhibition of amorphous drug crystallization in amorphous SDDS systems is possible due to the molecular interaction between drugs and excipients that occurs in those systems [30,41]. Strong intermolecular interactions, especially hydrogen bonding interactions between Soluplus^®^ and FEL, might further lower the molecular mobility of FEL and delay recrystallization during storage. Nevertheless, assay results indicated that amorphous SDDS systems generated via different processes similarly stabilized the amorphous state of the drug, prompting us to conduct assays to understand and explore underlying mechanisms at the molecular level, as described below.

#### 3.3.2. Underlying Molecular Interaction Facilitates the Immediate Release of faSDDS Systems

**Figure 8:**
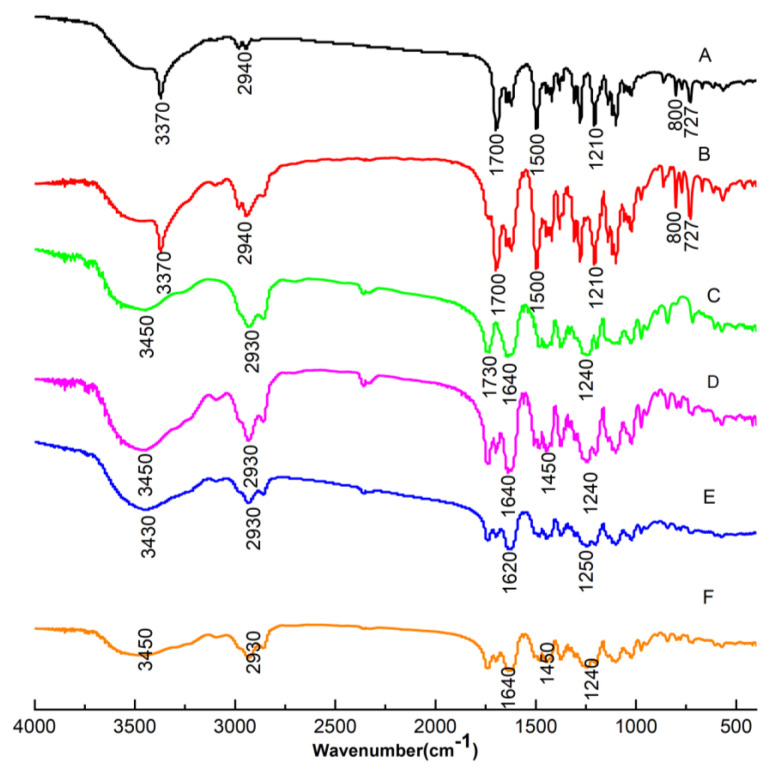
FT-IR spectra of samples including FEL (A), FEL_PM_ (B), Soluplus^®^ (C), faSDDS_SE_ (D), faSDDS_QC_ (E) and faSDDS_HME_ (F).

Commonly, the drug–polymer molecular interactions could result in identifiable FT-IR spectral changes. FEL contains hydrogen bond donor and acceptor groups in its molecular structure, with the FEL amine group participating in hydrogen bonding [42,43], while potential sites within the structure of Soluplus^®^ participate in hydrogen bonding as well. Importantly, any changes in peak position and shape in the FT-IR spectrum relative to the spectrum obtained for pure components would provide evidence of possible specific drugs-polymer molecular interactions, such as hydrogen bonding. Figure 8 displayed the results of Soluplus^®^, FT-IR spectra of pure FEL, physical mixtures and faSDDS systems. The FT-IR spectrum for FEL showed five strong feature peaks, including N–H stretch (3370 cm^−1^) and C=O double peaks (1700 cm^−1^), a benzene skeleton vibration peak (1500 cm^−1^) and two ester C-O-C peaks (1280 cm^−1^ and 1210 cm^−1^). The Soluplus^®^ spectrum contained four characteristic peaks, including one a O–H stretch peak at 3450 cm^−1^, a C=O ester peak (1730 cm^−1^), a caprolactam peak (1640 cm^−1^) and an ether C-O-C peak (1240 cm^−1^). For physical mixtures of FEL and Soluplus^®^, only characteristic peaks of FEL were clearly visible that were not influenced by the presence of Soluplus^®^. In all faSDDS systems, stretching peaks of NH groups (3370 cm^−1^) disappeared and only one weak wide peak was visible. These data implied that hydrogen bonds may have formed between FEL and Soluplus^®^ during the thermodynamic disordering process. Meanwhile, C=O peaks of all groups did not change, while blue-shifted peaks such as FEL benzene skeleton vibration peaks from 1500 cm^−1^ to 1510 cm^−1^, altered FEL ester C-O-C peaks from 1280 cm^−1^ to 1270 cm^−1^ and altered Soluplus^®^ ether C-O-C peaks from 1240 cm^−1^ to 1242 cm^−1^ were also observed.

Taken together, FT-IR results indicated that in faSDDS systems the –NH group of FEL participated in stronger hydrogen bonding interactions with acceptor groups of Soluplus^®^ than those present within molecules of the pure drug. In addition, the benzene structure of FEL may become embedded within hydrophobic chains of Soluplus^®^ via dispersion or polar forces acting during the faSDDS thermodynamic disordering process. Concomitantly, the involvement of the FEL –NH group in stronger hydrogen bonding with faSDDS acceptors as compared to acceptors within the pure drug indicated disruption of essential intermolecular hydrogen bonds (between FEL molecules) that are required for the formation of the FEL 3D crystal lattice conformation. Thus, this molecular interaction may have played a crucial role in the conversion of crystalline FEL to an amorphous form in faSDDS systems fabricated using HEM and other techniques by stabilizing the amorphous form, which is favorable to promote immediate release. However, despite the enhanced molecular interactions occurring in faSDDS compared to physical mixtures, no significant differences were observed in the SDDS systems prepared by HME and other discontinuous techniques.

#### 3.3.3. Soluplus^®^ in faSDDS Can Inhibit FEL Crystallization from a Supersaturated State and Improve Immediate Release

**Figure 9:**
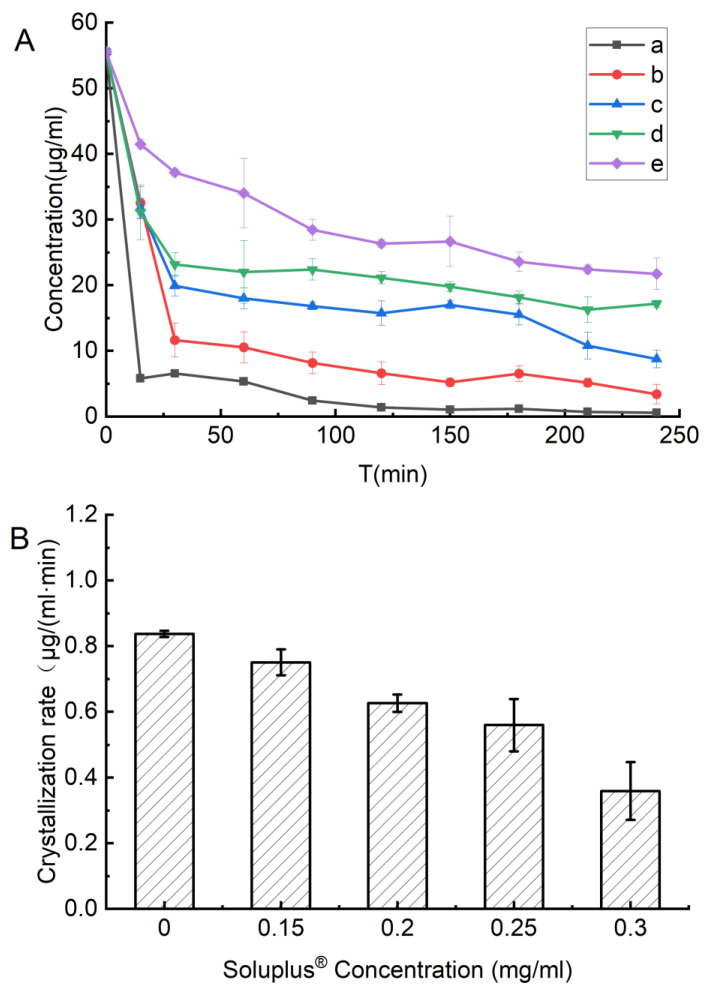
(**A**) Inhibition effects of Soluplus^®^ on the recrystallization of FEL from a supersaturated concentrated solution (55.56 μg/mL) at 37 °C in the PBS (pH 6.8) (a) and mixed with Soluplus^®^ of different concentration of 0.15 (b), 0.2 (c), 0.25 (d), or 0.3 mg/mL (e). (**B**) FEL crystallization rate at different Soluplus^®^ concentrations. Standard deviation expressed by error bars (*n* = 3).

Inhibitory influences of Soluplus^®^ on crystallization of FEL in the supersaturated solution were evaluated by adding a concentrated solution of FEL in methanol (50 mg in 0.3 mL) to PBS solutions (pH 6.8) containing pre-dissolved polymers followed by examination of drug solution concentrations as a function of time. Figure 9 and Table 2 show results obtained for polymer solution concentrations of 0, 0.15, 0.2, 0.25 and 0.3 mg/mL. The supersaturated FEL would crystallize and precipitate rapidly when there was no pre-dissolved Soluplus^®^ (Figure 9). By contrast, addition of Soluplus^®^ led to increased FEL concentration in solution, especially for higher amounts of pre-dissolved Soluplus^®^, which markedly inhibited FEL crystallization from a supersaturated state. Specifically, inhibition effects of different concentrations of pre-dissolved Soluplus^®^ on FEL crystallization were presented as concentration dependent manner in which it gradually diminished (0.3 mg/mL showed the highest but no effects was observed in none Soluplus^®^ group).

The half-times of FEL crystallization against Soluplus^®^ are displayed in Table 2. When there was no pre-dissolved Soluplus^®^, FEL showed a half-time crystallization of 8.38 min, indicating that FEL tended to return to a stable crystalline state in the absence of inhibitors. By contrast, the time of FEL crystallization could be delayed by addition of pre-dissolved Soluplus^®^, brought in a higher half-time of FEL, this correlation performed as concentration dependent manner, and FEL crystallization performed the longest half-time value (~89.68 min) when the concentration of pre-dissolved Soluplus^®^ was largest. In line with this result, an increase in added Soluplus^®^ reduced the FEL crystallization rate from 0.84 μg/(mL·min) without Soluplus^®^ to 0.36 μg/(mL·min) for a concentration of Soluplus^®^ of 0.3 mg/mL (Figure 9B). Meanwhile, FEL molecular structure was maintained in solution for the longest period of time when FEL was present in greatest excess.

The anti-crystallization mechanism can be explained by classical nucleation theory as described in Equation (4) [44].

(4) $$J=\mathrm{Aexp}\left[-\frac{16\pi \gamma^{3}\upsilon^{2}}{3k^{3}T^{3}\left(lnS\right)}\right]$$

where J represents the nucleation rate; *A* denotes a constant; *γ* represents the interfacial tension; *ν* represents the molecular volume; *k* represents the Boltzmann constant; *T* represents temperature and *S* represents supersaturation degree. From Equation (4), it is obvious that the nucleation rate of crystallization depends on the degree of supersaturation. When the FEL concentrated solution was injected into solutions without or with different Soluplus^®^ concentrations, crystallization occurred to different degree. When no polymer was present, rapid crystallization was observed. For lower pre-dissolved Soluplus^®^ concentrations (Figure 9), the FEL concentration decreased quickly from the initial 55.56 to 5.81 μg/mL in 15 min. However, in the presence of Soluplus^®^ (0.3 mg/mL), the FEL concentration decreased only slightly to 41.46 μg/mL after 15 min then decreased slowly thereafter. This result indicated that Soluplus^®^ could maintain FEL in a supersaturated state and was effective at inhibiting or delaying crystallization of FEL, whereby inhibition capability increased with increasing amount of Soluplus^®^ added to the system.

#### 3.3.4. Self-Micellizing Soluplus^®^ Itself Could Solubilize FEL in a Favorable/Spontaneous Manner and Enhance Immediate Release in faSDDS Systems

**Figure 10:**
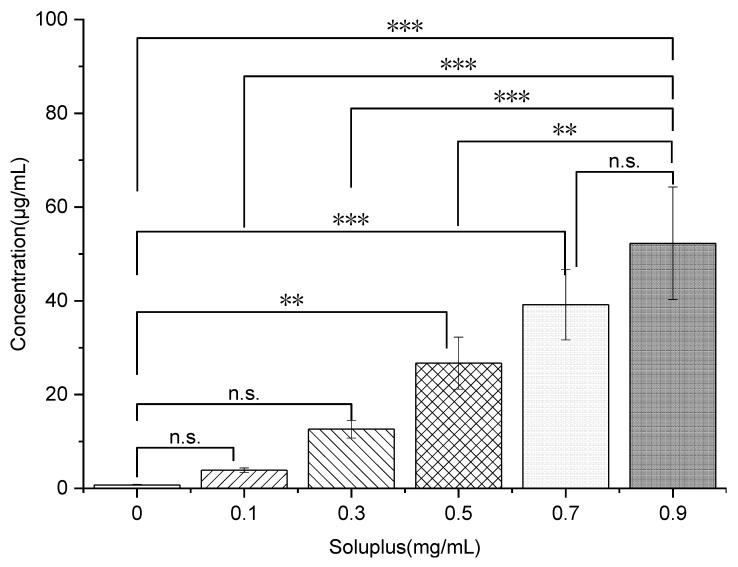
Phase solubility diagram of FEL in PBS (pH 6.8) mixed with the pre-dissolved polymer Soluplus^®^ at different concentrations (from 0.1 to 0.9 mg/mL) at 37 °C. ** *p* < 0.01, *** *p* < 0.001. Standard deviation expressed by error bars (*n* = 3).

The results show the phase solubility of FEL with different concentrations from 0 to 0.9 mg/mL when mixed with PBS (pH6.8) containing pre-dissolved Soluplus^®^ (Figure 10). The solubility of FEL was ~0.74 μg/mL when there was no Soluplus^®^ (Figure 10), while FEL phase solubility increased significantly (*p* < 0.01) as pre-dissolved Soluplus^®^ concentration increased. Notably, the highest phase solubility of FEL (~52.27 μg/mL) could be supported by Soluplus^®^ at a high concentration of 0.9 mg/mL, which measured about 70-times higher relative to samples containing no Soluplus^®^.

Phase-solubility tests revealed different parameters which showed that drug solubility was linear increased with the increasing carrier level (R^2^ = 0.9911). Similar effects have been reported on several other drugs and water-soluble carriers in previously published articles [45,46], and the possible reasons were mostly due to a co-solvent effect of the carrier and/or the formation of weakly soluble complexes. Meanwhile, studies revealed that the interaction between hydrophilic carriers and drug were mainly dependent on electrostatic or other types of forces including hydrogen bonds.

According to the theory of Equation (5) [47], the Gibbs free energy of transfer ($\Delta G_{tr}^{o}$) was tested, with results summarized in Table 3.

(5) $$\Delta G_{tr}^{o}=-2.303RT\cdot \log\frac{S_{o}}{S_{s}}$$

whereas the ratio of drug solubility in the presence/absence of Soluplus^®^ was marked by *S_o_*/*S_s_*, the molar gas constant was marked by R, the Gibbs free energy of transfer was marked by $\Delta G_{tr}^{o}$, and the selected experimental temperature was marked by *T*. FEL solubilization was obviously supported by the self-micellizing Soluplus^®^, since negative values for $\Delta G_{tr}^{o}$ were obtained for FEL that ranged from −4.06 to −10.47 KJ/mol in the presence of Soluplus^®^ that decreased with increasing carrier concentration. This result revealed that the process of FEL transfer from PBS solution to carrier solution became more favorable and spontaneous at higher carrier concentrations, thus demonstrating that Soluplus^®^ could act as crystallization inhibitor while also solubilizing poorly water-soluble FEL.

**Figure 11:**
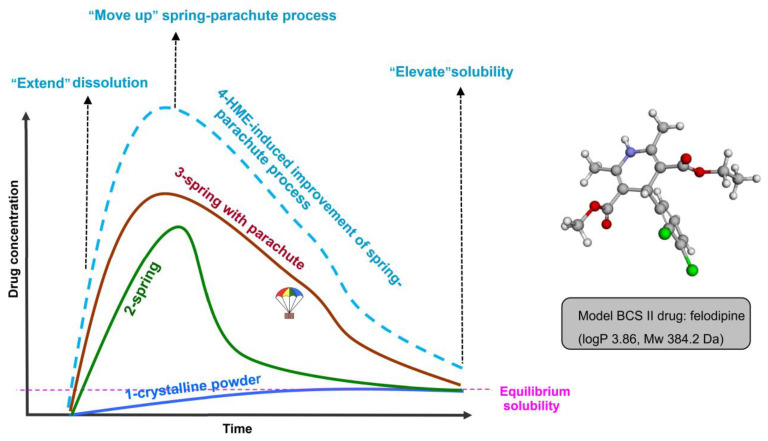
Illustration showing HME-induced improvement of supersaturable immediate-release at initial/evolved/terminal three-stage in faSDDS_HME_ systems containing the model BCS II drug, felodipine: 1 crystalline powder; 2 spring process without parachute process induced by pure amorphous drug; 3 spring-parachute process induced by amorphous faSDDS approach; 4 HME-induced improvement of spring-parachute process of faSDDS systems.

Finally, as summarized in Figure 11, faSDDS prepared by the HME method optimizes the immediate release behavior of SDDS by extending initial release at the initial dissolution stage, moving up the evolved release at “spring-parachute” process, and elevating terminal release at terminal stage. A superior amorphous SDDS system containing the BCS II antihypertensive drug felodipine was generated jointly employing HME technique and self-micellizing polymer. The study clarifies the advantage of HME-based process to trigger amorphization and extend multi-stage supersaturable immediate release, and is helpful for the development of superior amorphous faSDDS systems when employing self-micellizing polymer Soluplus^®^.

## 4. Conclusions

The exploitation of amorphous forms presents a frequently used approach to achieve supersaturation (“spring”) in supersaturating drug-delivery systems (SDDS). Because of inherently metastable nature of the state, amorphous active pharmaceutical ingredients (APIs) tend to return to stable crystalline form and result in phase transformations during immediate release in a liquid environment as well as storage in a solid environment, which offsets any advantage of amorphization and supersaturation. Due to this non-equilibrium state, the maintenance of the state (“parachute”) is often manipulated by introducing polymers. This is extremely important for the maintenance and even the extension of supersaturation (so-called “spring-parachute” process) during immediate release period. Comprehensive analysis of these critical release behaviors is crucial and associated with initial release after dosing and dissolution, evolved release during longer dissolution period for the biological absorption, and terminal release at the end of immediate release, especially when employing new techniques or polymers matched with a particular API. Our investigation aims to fabricate hypotensive supersaturating solid dispersion systems (called faSDDS_HME_) containing a particular BCS II drug, namely felodipine, when coordinately employing HME technique and self-micellizing polymer Soluplus^®^. Meanwhile, controlled SDDSs including faSDDS_SE_ and faSDDS_MC_ were developed based on solvent evaporation and microwave-cooling methods. faSDDS_HME_, faSDDS_SE_ and faSDDS_MC_ were characterized via SEM, and PXRD and for the establishment of their amorphization. Tailored initial/evolved/terminal three-stage supersaturable immediate-release behaviors were further clarified and possible mechanisms were explored. HME resulted in the highest initial release concerning faSDDS_HME_ according to dissolution assay, mainly due to HME-triggered superior supersaturation. During the evolved release period, HME-induced amorphization resulted in highly extended “spring-parachute” process due to the optimal duration of supersaturation generated. Solubility improvement in HME-formed systems showed that HME led to the strongest terminal drug release. For release mechanisms associated with HME, molecular interaction is not the likely dominant mechanism responsible for improved immediate release of faSDDS resulting from HME based on FT-IR analysis. However, it is possible that HME-associated effects were due to other distinctive molecular mechanisms that improved the properties of amorphous SDDS systems and suppressed crystallization of internal amorphous APIs warranting further study. For release mechanisms involved with the polymer Soluplus^®^, various aspects including molecular interaction, Noyes–Whitney equation, nucleation theory and Gibbs free energy of transfer ($\Delta G_{t}^{o}$) were explored. Soluplus^®^ was shown to be capable of inhibiting drug recrystallization, and solubilizing the drug spontaneously, leading to the improved molecular interactions in all faSDDS systems. Based on determinations of ${\Delta G}_{tr}^{o}$, the self-micellizing polymer Soluplus^®^ had a high Gibbs free energy of transfer that enabled the polymer to solubilize FEL in an instantaneous and spontaneous manner. The FT-IR analysis suggested that enhanced molecular interactions can be observed in all API-polymer-formed faSDDS systems and were possibly responsible for the improved supersaturable immediate release during initial/evolved/terminal stages through observations on the change of characteristic functional groups. These mechanisms were mainly responsible for the improvement in supersaturation-triggered multi-stage immediate release resulting from HME or self-micellizing polymer. This work provides a deeper insight on multi-stage supersaturable immediate-release behaviors and related release mechanisms for particular hypotensive SDDS formulations when synergistically employing the HME-based continuous manufacturing technique and self-micellizing strategy. The study also provides support for the transfer of HME-based hypotensive self-micellizing supersaturating delivery systems from the research stage to pharmaceutical application.

## Figures and Tables

**Table 1:** Quantification parameters of the “spring-parachute” process for different FEL-related samples.

Samples	AUC_spring-parachute_ (μg·h/mL)	T_max _ (h)	C_max_ (μg/mL)	C_36h _ (μg/mL)
FEL	24.80	15	1.14	0.76
FEL_PM_	27.01	14.5	1.39	0.77
FEL_PA_	36.34	16.5	1.62	0.96
faSDDS_SE_	58.41	6.5	2.23	1.42
faSDDS_QC_	48.63	12	2.15	1.17
faSDDS_HME_	70.52	7.5	2.65	1.70

**Table 2:** Half-time (T_1/2_) of the recrystallization of crystalline FEL from a supersaturated concentrated solution (55.56 μg/mL) at 37 °C in the mixed solution of PBS (pH6.8) without/with pre-dissolved Soluplus^®^ (0.15, 0.2, 0.25 and 0.3 mg/mL).

Concentration (mg/mL) of Pre-dissolved Soluplus^®^	Initial FEL Concentration (μg/mL) in Supersaturated Solution	Half-Time (min) of FEL Crystallization from a Supersaturated Sate
0	55.56	8.38 ± 0.11
0.15	55.56	35.08 ± 0.41
0.20	55.56	44.41 ± 1.84
0.25	55.56	50.40 ± 7.73
0.30	55.56	89.68 ± 6.08

**Table 3:** The Gibbs free energy of transfer ($\Delta G_{tr}^{o}$) of FEL in PBS (pH 6.8) with pre-dissolved Soluplus^®^ concentrations ranging from 0.1 to 0.9 mg/mL. Standard deviation expressed by error bars (*n* = 3).

Concentration (mg/mL) of Soluplus^®^	Drug	$\Delta G_{tr}^{o}$
0.1	FEL	−4.06 ± 0.31
0.3	FEL	−6.98 ± 0.39
0.5	FEL	−8.82 ± 0.55
0.7	FEL	−9.76 ± 0.50
0.9	FEL	−10.47 ± 0.62

## Data Availability

Data are presented herein.
